# An apical actin-rich domain drives the establishment of cell polarity during cell adhesion

**DOI:** 10.1007/s00418-012-0965-9

**Published:** 2012-05-29

**Authors:** Federico Galvagni, Cosima Tatiana Baldari, Salvatore Oliviero, Maurizio Orlandini

**Affiliations:** 1 Dipartimento di Biotecnologie, Università degli Studi di Siena, Via Fiorentina 1, 53100 Siena, Italy; 2 Dipartimento di Biologia Evolutiva, Università degli Studi di Siena, Via Aldo Moro 2, 53100 Siena, Italy; 3 Human Genetics Foundation (HuGeF), Via Nizza 52, 10126 Turin, Italy

**Keywords:** Cell spreading, Moesin, VE-cadherin, Src, Cytoskeleton, Endothelial cell

## Abstract

One of the most important questions in cell biology concerns how cells reorganize after sensing polarity cues. In the present study, we describe the formation of an actin-rich domain on the apical surface of human primary endothelial cells adhering to the substrate and investigate its role in cell polarity. We used confocal immunofluorescence procedures to follow the redistribution of proteins required for endothelial cell polarity during spreading initiation. Activated Moesin, vascular endothelial cadherin and partitioning defective 3 were found to be localized in the apical domain, whereas podocalyxin and caveolin-1 were distributed along the microtubule cytoskeleton axis, oriented from the centrosome to the cortical actin-rich domain. Moreover, activated signaling molecules were localized in the core of the apical domain in tight association with filamentous actin. During cell attachment, loss of the apical domain by Moesin silencing or drug disruption of the actin cytoskeleton caused irregular cell spreading and mislocalization of polarity markers. In conclusion, our results suggest that the apical domain that forms during the spreading process is a structural organizer of cell polarity by regulating trafficking and activation of signaling proteins.

**Electronic supplementary material:**

The online version of this article (doi:10.1007/s00418-012-0965-9) contains supplementary material, which is available to authorized users.

## Introduction

Cell polarity is the asymmetric organization of most of the physical structures of the cell. Although different cell types and organisms show cellular asymmetries, establishment, maintenance, and transduction of cell polarity are characterized by common general steps: a polarity cue, asymmetric accumulation of polarity factors, and reorganization of the cytoskeleton.

In endothelial cells, the establishment of apical–basal polarity is a fundamental requirement for lumen formation in different types of vessels. However, whereas much is known about the molecular pathways controlling epithelial lumen morphogenesis (St Johnston and Ahringer 2010), only recent in vivo experiments, together with previous in vitro observations, have highlighted the importance of specific proteins in vascular tube formation (Iruela-Arispe and Davis 2009; Herbert and Stainier 2011). During arteriolar lumen morphogenesis, direct interaction of endothelial cells with the extracellular matrix (ECM) through β1 integrin was identified as an initial and critical cue for asymmetric distribution of regulatory proteins, among which partitioning defective 3 (Par3) plays a key role in the acquisition of apical–basal polarity (Zovein et al. 2010). Once endothelial cell polarity is established, β1 integrin and vascular endothelial cadherin (VE-cadherin) control the recruitment to apical membranes of the surface glycoprotein podocalyxin (PODXL). PODXL, which is able to generate apical-domain structures (Nielsen and McNagny 2008), connects with Moesin, a member of the Ezrin/Radixin/Moesin (ERM) family of actin-binding proteins (Strilic et al. 2009; Zovein et al. 2010). The apical accumulation of Moesin modulates mechanical properties in endothelial cells by recruiting filamentous actin (F-actin) and controlling cell polarity (Strilic et al. 2009; Wang et al. 2010). Expansion of the blood vessel lumen seems to involve VEGF signaling and endothelial vacuole trafficking (Strilic et al. 2009; Zovein et al. 2010). However, in spite of the molecular polarity pathways identified, it remains unclear how cortical polarity controls the reorganization of the actin and microtubule cytoskeletons and the polarization of trafficking pathways.

Work in signal transduction has now pointed out the importance of specialized membrane domains in organizing the spatial distribution of ligands, receptors and downstream components to bring them in close proximity (Lajoie et al. 2009). To organize and maintain specialized membrane domains, cells have to coordinate processes at the cell surface with those occurring in the underlying cortical cytoplasm and cytoskeleton (Fehon et al. 2010). Several studies have implicated Moesin, the most abundant ERM protein in endothelial cells, as key regulator of the structure and function of specific domains in the cell cortex (Fehon et al. 2010). In addition to its well-known role in organizing the cellular apical membranes, recent work indicates that Moesin coordinates the establishment of cortical polarity during cell spreading of melanoma cells (Estecha et al. 2009), and also regulates membrane-associated signaling pathways (Neisch and Fehon 2011). However, how cortical domains are formed and maintained, and how signaling complexes are assembled and distributed as cells first contact the ECM remain to be elucidated.

Here, we identified a cellular domain with a *bud*-like structure on the apical surface of endothelial cells that are adhering to ECM. The apical bud represents an initial step to drive apical–basal polarity and protein trafficking during the initial phases of cell attachment. We show that key regulators of endothelial cell polarity localize to this domain and that this formation is Moesin- and actin-dependent because Moesin silencing or drug depolymerization of actin cytoskeleton cause apical bud impairment. Loss of apical bud affected not only regular cell spreading and morphology, but also sorting of polarity markers. Moreover, apical bud integrity was found to be instrumental for the control of signaling pathways involved in the regulation of cell polarity.

## Materials and methods

### Cell culture

Human umbilical vein endothelial cells (HUVEC) were isolated from umbilical cords collected from uncomplicated pregnancies, cultured on gelatin-coated Petri dishes in M199 culture medium containing 20 % fetal bovine serum, 50 units/mL penicillin–streptomycin, 10 units/mL heparin, and 100 μg/mL brain extract, and used in passages 3–6. Bovine coronary venular endothelial cells (CVEC) from Marina Ziche (University of Siena, Italy) were grown on gelatin-coated plastic dishes in the same culture medium used for HUVEC. Immortalized mammary epithelial cells (IMEC) were provided by Myles Brown (Dana-Farber Cancer Institute, Boston, MA, USA) and cultured in MEBM culture medium with MEGM supplements (Lonza, Basel, Switzerland). Mouse embryonic fibroblasts (MEF) were obtained and cultured as previously described (Orlandini and Oliviero 2001). The BALB/c, F9, Hs578T, Saos-2, HeLa, MCF7, and A549 cell lines (ATCC, Manassas, VA, USA) were cultured using standard conditions.

### Spreading and cell division analyses

For spreading analysis, cells were trypsinized, resuspended in complete medium, plated on sterile coverslips coated with an adhesive substrate, and allowed to spread for various times. Cells at specific phases of spreading were fixed, labeled, and imaged by confocal microscopy.

To analyze dividing cells, exponentially growing HUVEC were trypsinized and immediately replated subconfluent on coverslips coated with vitronectin. To take off floating cells, 2 h after replating, culture medium was removed, cells were washed with PBS, and fresh medium was added. 20 h after medium removal cells were fixed, stained, and imaged by confocal microscopy.

### Reagents and antibodies

The following adhesive substrates were dissolved in PBS and used for coating (2 h at 37 °C): human vitronectin (1 μg/mL) from Invitrogen (San Diego, CA, USA), rat collagen (from tail tendon, mainly type I collagen, 10 μg/mL) from Roche Diagnostics (Mannheim, Germany), and porcine gelatin (1 %), mouse laminin (from Engelbreth-Holm-Swarm sarcoma, 10 μg/mL), human fibronectin (10 μg/mL), and human type IV collagen (10 μg/mL) from Sigma-Aldrich (St Louis, MO, USA). Poly-Prep slides coated with poly-l-lysine were from Sigma-Aldrich.

Latrunculin B (Calbiochem, La Jolla, CA, USA) was used at the dose of 0.5 μM, which has been shown to fully disrupt actin microfilament integrity in human capillary endothelial cells (Huang et al. 1998). Nocodazole (Sigma-Aldrich) was used at the concentration of 20 μM. In spreading analysis, cells were pretreated for 30 min with latrunculin B, or for 1 h with nocodazole. Then cells were trypsinized, replated on glass coverslips in complete culture medium containing the drug and fixed at different time points. The Src family tyrosine kinase inhibitor PP2 was purchased from Calbiochem.

The following primary antibodies were used: mouse anti-Podocalyxin (R&D Systems, Minneapolis, MN, USA); rabbit anti-Moesin and rabbit anti-phospho-Y416 Src (Cell Signaling, Danvers, MA, USA); mouse anti-phosphotyrosine (clone 4G10) and rabbit anti-Par3 (Millipore, Billerica, MA, USA); mouse anti-acetylated α-tubulin and mouse anti-β-actin (Sigma-Aldrich); rabbit anti-phosho-Y951 Flk-1 (VEGFR-2) receptor, rabbit anti-caveolin-1, rabbit anti-phospho-Moesin, mouse anti-Rab11, rabbit anti-VEGFR-2 (sc-504), and goat anti-VE-cadherin (Santa Cruz Biotechnology, Santa Cruz, CA,USA); rabbit anti-Centrin 1 and rabbit anti-Giantin (Abcam, Cambridge, UK); mouse anti-Rab5 (BD Biosciences, Franklin Lakes, NJ, USA); mouse anti-transferrin receptor (hybridoma OKT9) was generously provided by A. Alcover.

### Immunofluorescence microscopy

Cells seeded onto coated glass coverslips were fixed in 3 % paraformaldehyde in PBS for 15 min at room temperature. Samples were permeabilized with 0.5 % Triton X-100 in PBS, blocked with 1 % bovine serum albumin in PBS, and incubated for 1 h at 37 °C with the primary antibodies. The secondary antibodies used were conjugated with Alexa Fluor-488 and Alexa Fluor-568 (Invitrogen). Alexa Fluor-488, -568, and -647 phalloidin (Invitrogen) were used for F-actin labeling. TOPRO-3 (Invitrogen) was used for nuclear staining. Slides were mounted in Mowiol 4-88 (Calbiochem) and fluorescent images were captured using a Leica TCS SP2 laser scanning confocal microscope. To better dissect the phases of spreading, some cells were shown as lateral views, corresponding to single *xz* planes. These images were processed from serial *z*-stacks acquired at ~0.115-μm intervals by using the Leica Confocal Software LCS 2.61 build 1537.

### RNA interference-mediated knock down of VEGFR-2 and Moesin

Silencing experiments were performed using pLKO.1 retroviral vectors from TRC lentiviral shRNA libraries expressing specific shRNAs for human VEGFR-2 (Open Biosystems; clone ID: TRCN0000001685, referred to as 85, and TRCN0000001686, referred to as 86), and Moesin (Sigma-Aldrich; clone ID: TCRN0000344732, validated by the company and referred to as M32). Recombinant lentiviruses were produced and used for infection experiments as previously described (Orlandini et al. 2008). Immunoblotting analyses were performed as previously described (Orlandini et al. 2008).

## Results

### An actin-rich domain forms at the apical surface of endothelial cells during spreading

**Fig. 1:**
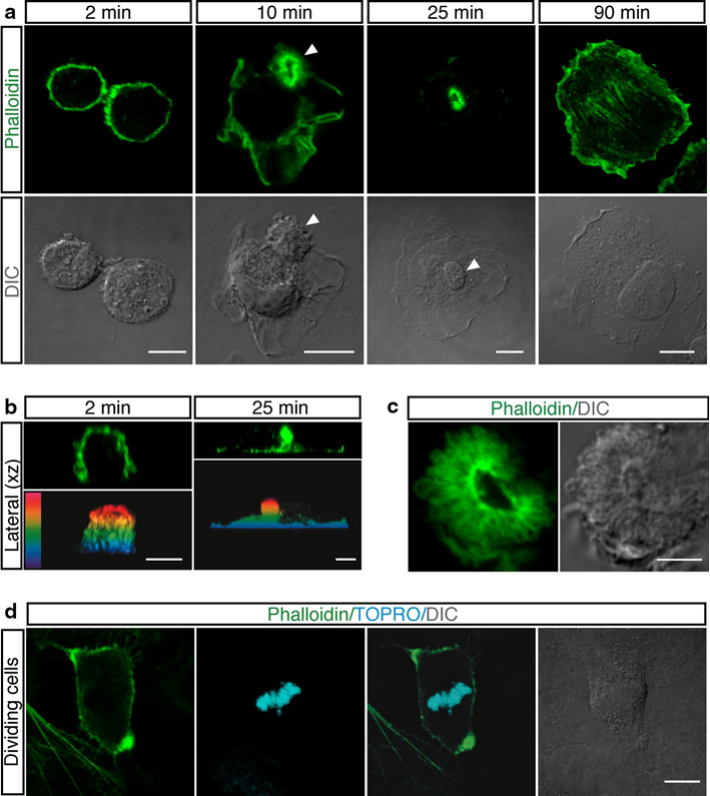
Spreading of round endothelial cells is characterized by the formation of an actin-rich domain in the cellular apical membrane. **a** HUVEC were trypsinized and resuspended in complete medium. To examine different phases of spreading, cells were allowed to settle on vitronectin-coated coverslips, fixed at the indicated times after plating and stained with phalloidin (*green*). *Arrowheads* indicate the actin-rich domain. *Scale bars* represent 8 μm. **b** Periodic *z*-series of HUVEC treated as in **a** were acquired at 2 and 25 min after plating to generate vertical sections (lateral *xz*). The same images are shown as color-coded projections such that the basal cell surface, which adheres to the substrate is violet and the apical cell surface is red. *Scale bars* 6 μm. **c** Details of an actin-rich domain formed on the apical surface of spreading HUVEC treated as in **a**. *Scale bar* 4 μm. **d** HUVEC were plated on coverslips coated with vitronectin. After 2 h, the culture medium was removed, cells washed with PBS and fresh medium added. 20 h after medium removal, samples were stained for F-actin (*green*) and DNA (*blue*). Mitotic cells display actin-rich domains located at the cellular edges. *Scale bar* is 8 μm. DIC images are shown

Following endothelial cell attachment on vitronectin-coated surfaces, we observed that every cell displayed a membrane domain enriched in F-actin. Since an actin-rich domain was found to be involved in orienting apical–basal polarity in intestinal epithelial cells (Baas et al. 2004), we further investigated the formation and role of this domain in primary endothelial cells. Exponentially growing HUVEC were trypsinized, allowed to adhere on vitronectin-coated surfaces, and analyzed by immunofluorescence at different degrees of cell spreading and flattening against the substrate. Consistent with previous work on melanoma cells (Estecha et al. 2009), we observed that when endothelial cells contacted the substrate and began to adhere, they underwent transition from round to hemispheric shape and F-actin mainly localized to the periphery of the cells (Fig. 1a, 2 min; b, top left). At this early stage of attachment, endothelial cells displayed blebs on the cell surface and an actin-free region at the attaching edge (Fig. 1b, 2 min; animation Online Resource 1). As cell spreading increased, cells formed an actin-rich bud, which was maintained during the entire duration of the spreading phase and disappeared when cells were fully flattened on the substrate (Fig. 1a). The actin-rich domain was localized at the top of the cell (Fig. 1b, 25 min; animation Online Resource 2). To better visualize the apical position of the actin bud, lateral views of endothelial spreading cells were imaged using a color-coded projection indicating basal–apical position (Fig. 1b; animation Online Resource 3). At higher magnification, the apical bud showed radial symmetry with an actin-negative core from which F-actin branched out (Fig. 1c).

**Fig. 2:**
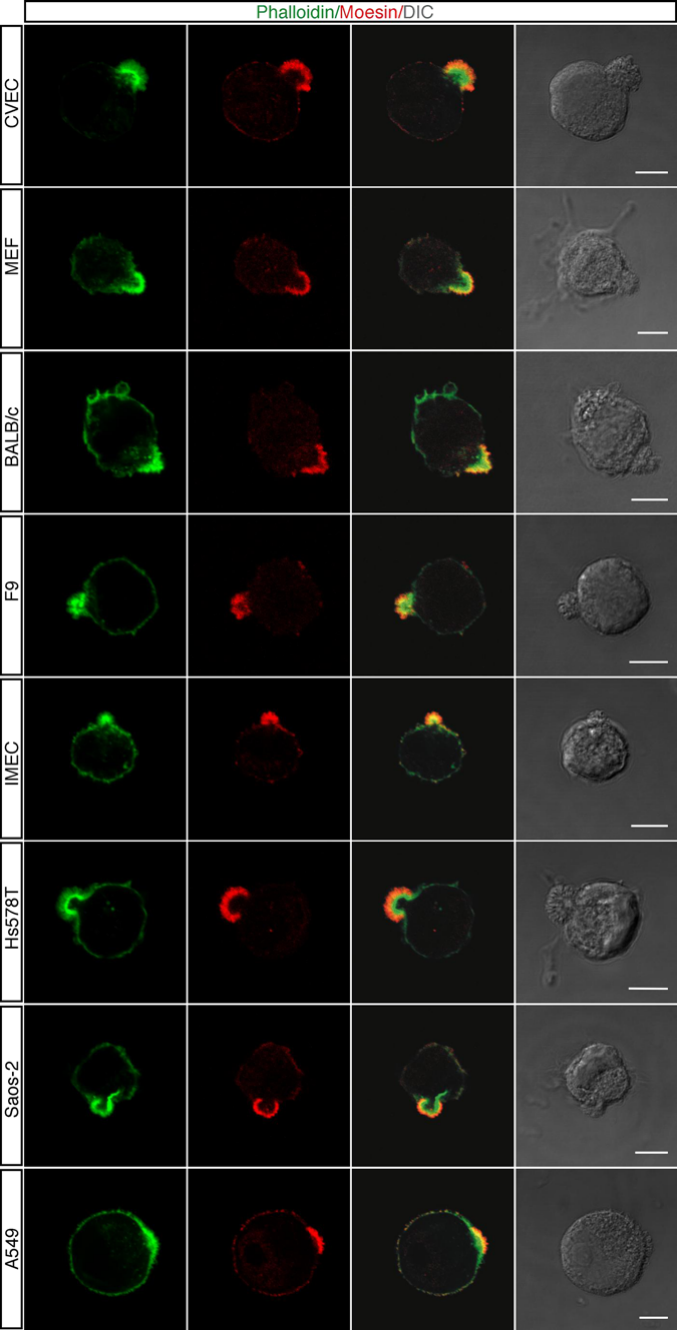
Different cell types display an apical domain enriched in F-actin and Moesin during adhesion to ECM. Cells were grown using standard conditions, detached from the plate, resuspended in complete medium and plated on vitronectin. Cells were analyzed during the initial phase of spreading using phalloidin (*green*) and anti-Moesin antibodies (*red*). DIC images of stained cells are shown. *Scale bars* represent 7 μm

At mitosis entry, actin reorganization at the plasma membrane induces cortical stiffness and cell rounding (Matzke et al. 2001) then cells divide and spread onto the substrate. To assess whether postmitotic cells formed apical actin-rich domains, we plated exponentially growing HUVEC on vitronectin, removed floating cells by washing, and then analyzed dividing cells. Mitotic cells were identified by chromatin condensation as well as overall changes in the cell morphology. As shown in Fig. 1d, mitotic cells exhibited actin-rich domains located at the cellular edges comparable to the apical buds observed in adhering cells. In our experiments, HUVEC were plated after trypsin treatment. Since proteolytic activity may harm cultured cells and have a remarkable adverse effect on cell physiology (Huang et al. 2010), endothelial cells were released from the substrate by EDTA treatment and resuspended as single cells in culture medium. During spreading, these cells formed regular apical actin domains (Online Resource Fig. S1a). Moreover, as adhesive substrates may have different effects on the stabilization of the cytoskeletal network (Chen et al. 1997), we analyzed the impact of several ECM proteins on apical bud formation. In addition to vitronectin, spreading of endothelial cells on gelatin-, laminin- fibronectin-, type I collagen-, type IV collagen-, and poly-l-lysine-coated substrates induced the formation of actin buds on the cell membranes (Online Resource Fig. S1b). Importantly, in growth factor-free culture conditions every endothelial cell displayed on its membrane surface an actin-rich bud during the adhesion phases (Online Resource Fig. S1c). Furthermore, to determine whether besides endothelial cells other cell types form an apical domain enriched in actin during initial attachment, we extended our analysis to different mammalian cell types. Imaging of cells fixed at early attachment times showed that the apical actin-rich domain formed not only in CVEC, but also in MEF and BALB/c 3T3 fibroblasts, murine F9 teratocarcinoma cells, human IMEC, human osteosarcoma cells (Saos-2), human breast cancer cells (Hs578T), and human lung adenocarcinoma epithelial cells (A549) (Fig. 2). Depending on the cell type, apical domains were different in size and morphology. Human breast cancer cells (MCF7) and cervical cancer cells (HeLa) did not show apical actin domain formation during the spreading phases (data not shown). Collectively, our data show that the presence of a domain enriched in F-actin on the apical cortex of round cells during attachment to the substrate is a general phenomenon, suggesting a role for this domain in cell polarization.

### VE-cadherin, activated Moesin and Par3 redistribute to the apical bud following initial spreading

**Fig. 3:**
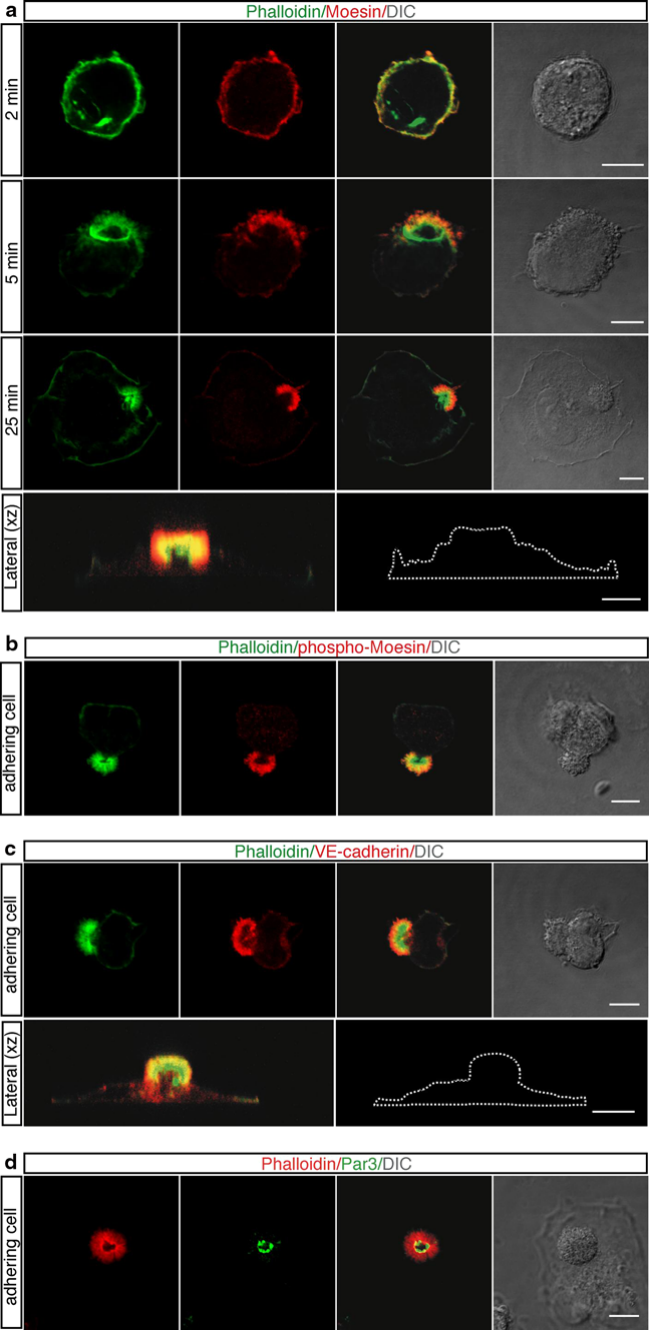
Following endothelial cell adhesion to ECM, activated Moesin and VE-cadherin redistribute to the apical bud. HUVEC were trypsinized, resuspended in complete medium, plated on vitronectin, and allowed to spread. **a** At the indicated times after plating cells were fixed and stained with phalloidin (*green*) and anti-Moesin antibodies (*red*). Moesin localizes to the apical domain. A vertical section (lateral *xz*) obtained from periodic *z*-series acquired at 25 min after plating of a spreading cell is shown. The corresponding cell boundary is indicated by a *white dotted line*. *Scale bars* represent 8 μm. **b** Cells attaching to the substrate were fixed and stained with phalloidin (*green*) and anti-phospho-Moesin antibodies (*red*). Activated Moesin localizes to the apical bud. *Scale bar* is 10 μm. **c** Spreading cells were fixed and stained with phalloidin (*green*) and anti-VE-cadherin antibodies (*red*). VE-cadherin is mainly distributed to the apical domain. Periodic *z*-series of a spreading cell were acquired at 20 min after plating to generate a lateral view (lateral *xz*). The corresponding cell boundary is indicated by a *white dotted line*. *Scale bars* represent 8μm. **d** Cells adhering to the substrate were fixed and stained with phalloidin (*red*) and anti-Par3 antibodies (*green*). Par3, a component of the Par polarity complex, localizes to the centre of the apical bud.* Scale bar* is 8 μm. DIC images of stained cells are shown

To assess whether the apical actin domain is involved in cell polarization of adhering cells, we focused on Moesin, VE-cadherin and Par3, which have been demonstrated to be required for endothelial apical–basal polarity in vitro and during embryonic development (Strilic et al. 2009; Lampugnani et al. 2010; Wang et al. 2010; Zovein et al. 2010). To study the dynamic distribution of Moesin, VE-cadherin and Par3 during cell spreading, endothelial cells were fixed and stained at different time points after plating onto vitronectin. F-actin and Moesin were detectable at the rounded cortex of the cells immediately after contact with the substrate (Fig. 3a, 2 min). As cell attachment progressed, Moesin polarized at the presumptive site of apical bud formation (Fig. 3a, 5 min), and when the apical bud was fully structured, Moesin was essentially confined to this domain (Fig. 3a, 25 min). To better visualize Moesin localization in the apical bud, a cross-sectional view through a spreading cell is shown (Fig. 3a, lateral *xz*). It has been demonstrated that Moesin exists in a closed conformation that is released by phosphorylation of its C-terminal domain (T558) with exposure of the F-actin-binding site (Fehon et al. 2010) and that phosphorylated Moesin causes cell rounding (Carreno et al. 2008; Kunda et al. 2008). Accordingly, Moesin was found to be phosphorylated on T558 not only in the rounded cell cortex of endothelial cells (data not shown), but also in the apical actin-rich domain of adhering cells (Fig. 3b), demonstrating that in the apical bud Moesin is active and able to link F-actin. Moreover, during cell adhesion diverse mammalian cells and postmitotic endothelial cells displayed Moesin accumulation in the actin-rich domains (Fig. 2, Online Resource Fig. S2). Interestingly, VE-cadherin had a pattern of localization similar to Moesin, being mainly concentrated in the apical domain of adhering endothelial cells and colocalizing with F-actin (Fig. 3c). To better visualize VE-cadherin distribution in the apical domain, a cross-sectional view through a spreading cell was shown (Fig. 3c, lateral *xz*). Importantly, Par3 localized to the inner side of the apical actin-rich bud of adhering endothelial cells (Fig. 3d), consistently with previous data showing Par3 able to directly bind VE-cadherin (Iden et al. 2006). Taken together, these results indicate that during early attachment of round endothelial cells to ECM, VE-cadherin, fully activated Moesin and Par3 become polarized and are mainly localized to the cortical bud in association with F-actin.

### The apical bud controls sorting of polarity proteins to other cellular domains

**Fig. 4:**
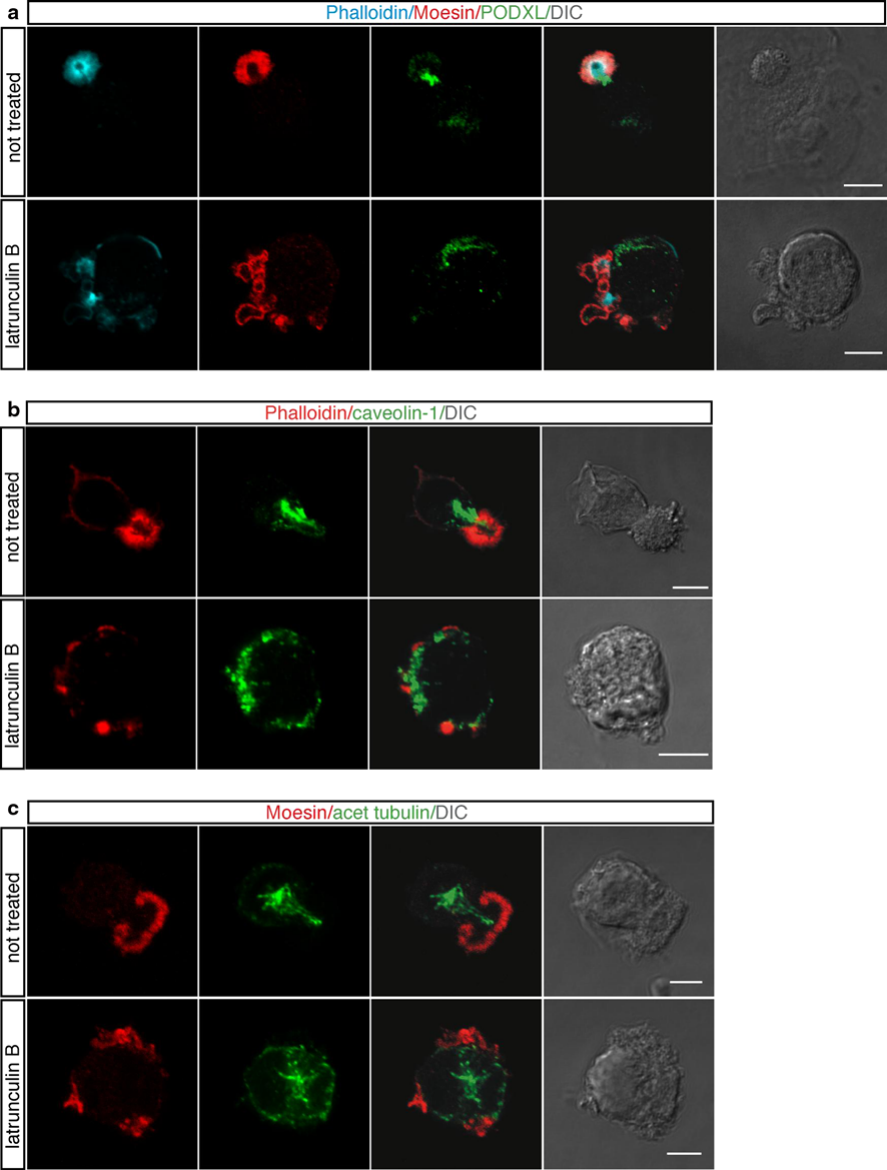
In spreading cells, polarity proteins are sorted towards the apical domain, whose positioning depends on actin. Endothelial cells were pretreated or not for 30 min with latrunculin B, then they were trypsinized, resuspended in complete medium and plated on vitronectin-coated surfaces in the presence or not of latrunculin B. **a** Cells were analyzed during the initial phase of spreading using phalloidin (*blue*), anti-Moesin (*red*), and anti-PODXL (*green*) antibodies. PODXL does not colocalize with Moesin on the cell external surface. *Scale bars* represent 6 μm. **b** Cells attaching to the substrate were labeled with phalloidin (*red*) and anti-caveolin-1 antibodies (*green*). Caveolin-1 exhibits a pattern of localization similar to PODXL. *Scale bars* 8 μm. **c** Spreading cells were stained with anti-Moesin (*red*) and anti-acetylated tubulin (*green*) antibodies. Acetylated microtubules extend out towards the apical bud. *Scale bars* are 5 μm. Following actin depolymerization PODXL, caveolin-1, and acetylated tubulin are randomly localized. DIC images of stained cells are shown

Since phosphorylated Moesin links F-actin to membrane proteins (Fehon et al. 2010), we asked whether other proteins involved in endothelial cell polarization localize to the apical domain. First, we examined PODXL, a protein belonging to the CD34-sialomucin family of cell surface glycoproteins (Nielsen and McNagny 2008). PODXL is potently targeted to the apical surface of endothelial cells where it binds Moesin and plays a functional role in generating a vascular lumen (Strilic et al. 2009). Interestingly, immunofluorescence analysis of endothelial cells at early times of spreading showed that PODXL did not colocalize with Moesin on the external surface but accumulated in the cytoplasmic side of the apical bud (Fig. 4a, upper panels and Online Resource Fig. S3a). The lateral view of a spreading cell visualizes internal localization of PODXL in the apical bud (Online Resource Fig. S3b, left panels). A similar pattern of localization was also observed with caveolin-1 (Fig. 4b, upper panels), another plasma membrane protein involved in endothelial cell polarization (Beardsley et al. 2005; Grande-Garcia and del Pozo 2008). Moreover, high-resolution immunofluorescence analyses showed PODXL and caveolin-1 distributed into discrete spots (Online Resource Fig. S3a, c) suggesting that in adhering cells the attempt to polarize requires an ordered sequence of events, including the formation of an actin-rich domain that guides redistribution of surface molecules to polarized domains via intracellular vesicles.

**Fig. 5:**
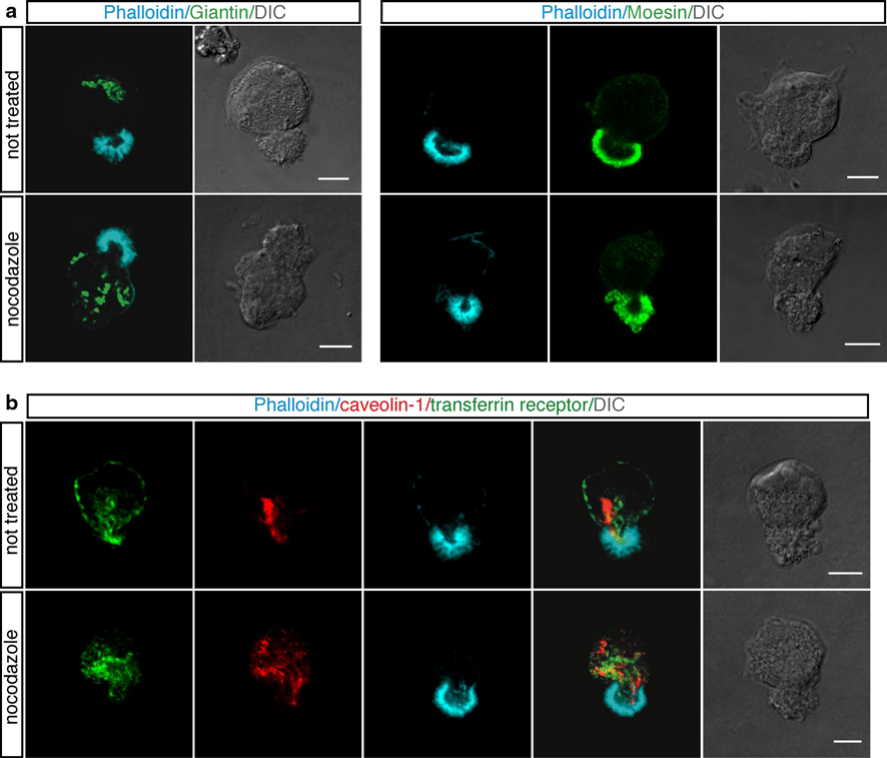
Nocodazole treatment impairs intracellular protein trafficking but not apical bud formation. HUVEC were pretreated or not for 1 h with nocodazole, then were trypsinized, resuspended in complete medium and plated on vitronectin-coated coverslips in the presence or not of nocodazole. **a** Cells were analyzed during the initial phases of attachment using phalloidin (*blue*) and anti-Giantin, a marker for the Golgi complex, or anti-Moesin antibodies (*green*). Cells exhibit regular apical buds after microtubule depolymerization. *Scale bars* represent 8 μm. **b** Endothelial cells were stained for transferrin receptor (*green*), a recycling endosomal marker, caveolin-1 (*red*) and F-actin (*blue*). Both the transferrin receptor and caveolin-1, which are localized along the centrosome-apical bud axis, are randomly distributed after nocodazole treatment. *Scale bars* represent 6 μm. DIC images of stained cells are shown

Since in polarizing cells, acetylated microtubules are oriented to support protein transport to areas of high need (Quinones et al. 2011), we analyzed acetylated tubulin distribution during endothelial cell spreading. Immunofluorescence analyses showed that the centrosome was located above the nucleus (Online Resource Fig. S4a) and that acetylated microtubules, anchored at the centrosome, extended out toward the inner area of the apical bud (Fig. 4c, upper panels), suggesting their involvement in protein trafficking towards the apical domain. The use of antibodies against Rab GTPases as markers for intracellular vesicular traffic allowed us to confirm the presence of a transport route along the centrosome-apical bud axis (Online Resource Fig. S4b). Because the oriented organization of the actin and microtubule cytoskeletons along the axis of polarity is a fundamental characteristic of cell polarity (Li and Gundersen 2008), we asked whether disruption of the microtubule and actin cytoskeletons could affect apical bud formation. To address this question, cells were let to settle on ECM in the presence of nocodazole, which interferes with the polymerization of microtubules, or latrunculin B, which disrupts microfilament organization. When the microtubule cytoskeleton was disrupted, the perinuclear organization of the Golgi apparatus was fragmented and dispersed throughout the cytoplasm as expected, however, no effects were seen on the formation of the apical bud and Moesin localization to this domain (Fig. 5a). Importantly, the transferrin receptor, a recycling endosomal marker, had a pattern of localization similar to PODXL and caveolin-1 along the centrosome-apical bud axis during the initial phases of spreading and treatment with nocodazole resulted in an altered cellular distribution of both the transferrin receptor and caveolin-1 (Fig. 5b). On the other hand, latrunculin B-treated cells displayed blebs and an irregular shape during the spreading process. Disruption of F-actin resulted in the impairment of apical bud structure and led to Moesin accumulation in multiple aberrant apical domains with different shape and size (Fig. 4a, c, bottom panels; Online Resource Fig. S3b, right panels). Moreover, following F-actin depolymerization PODXL, caveolin-1, and acetylated tubulin were randomly localized in adhering cells (Fig. 4a–c, bottom panels), and VE-cadherin was also mislocalized in several cytosolic regions (Online Resource Fig. S5a).

**Fig. 6:**
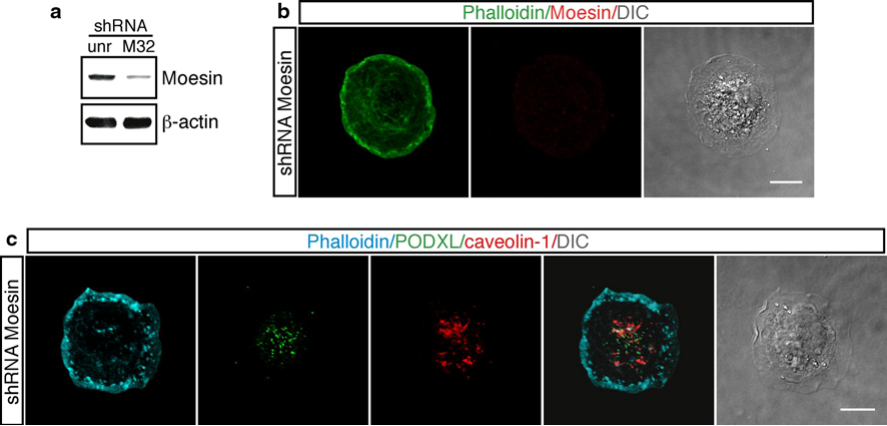
Effects of Moesin silencing in endothelial cells during the early phases of adhesion. **a** HUVEC were infected with a lentiviral vector expressing unrelated (unr) or Moesin (clone M32) shRNA. Cell extracts from HUVEC expressing shRNA were analyzed by western blotting using anti-Moesin and anti-β-actin antibodies. Cells infected with lentiviruses expressing Moesin shRNA show a reduced protein expression with respect to cells infected with a lentivirus expressing an unrelated shRNA. **b** Endothelial cells infected as in **a** were detached from the plate, seeded on vitronectin-coated coverslips, and fixed at different times after plating. Cells were stained for F-actin (*green*) and Moesin (*red*). Moesin-silenced cells do not form apical buds during spreading. *Scale bar* is 10 μm (**c**). Cells treated as in **b** were stained for F-actin (*blue*), PODXL (*green*) and caveolin-1 (*red*). PODXL and caveolin-1 are randomly distributed into the cell. *Scale bar* represents 8 μm. Representative adhering cells fixed after 8 min from plating are shown. DIC images of stained cells are shown

Since Moesin links F-actin we asked whether also Moesin was required for the formation of the apical bud. To address this question we performed RNA interference experiments by silencing Moesin in endothelial cells (Fig. 6a). Similar to previous work (Estecha et al. 2009), we observed that cells with reduced Moesin expression spreaded and flattened faster than control cells. Immunofluorescence analysis of Moesin-silenced cells fixed at the initial phases of adhesion showed that cells did not display apical buds on the cell membrane and that PODXL and caveolin-1 were randomly distributed throughout the cell (Fig. 6b, c). Collectively, these results suggest that, following cell adhesion, the microtubule network regulates the sorting of plasma membrane markers to other locations within the cell via the apical bud. In addition, impairment of the apical domain, whose structure is controlled by Moesin and the actin cytoskeleton architecture, hinders transport and distribution of polarity proteins.

### Signaling proteins are phosphorylated in the apical bud of spreading endothelial cells

**Fig. 7:**
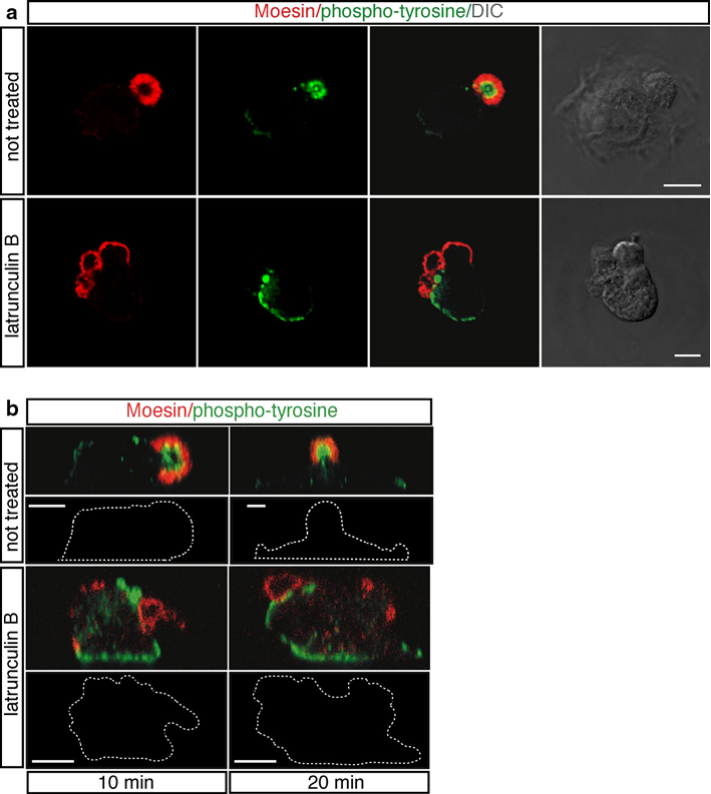
The core of the apical domain is a site of active signal transduction during spreading. HUVEC were pretreated or not for 30 min with latrunculin B, then were trypsinized, resuspended in complete medium and plated on vitronectin coat in the presence or not of the drug. **a** Cells were analyzed during early spreading using anti-Moesin (*red*) and anti-phosphotyrosine (*green*) antibodies. Tyrosine-phosphorylated proteins are concentrated in the inner side of the apical bud and after drug-treatment distribute throughout the cell. DIC images of stained cells are shown. *Scale bars* represent 6 μm. **b** Endothelial cells were stained as in **a**. Periodic *z*-series were acquired at 10 and 20 min after plating and the corresponding lateral views (*upper panels*) and *white dotted lines* indicating cell boundary (*bottom panels*) are shown. *Scale bars* represent 5 μm

The actin cytoskeleton has been demonstrated to play a key role in signal transduction, and different signaling proteins have been shown to associate directly to actin (Payrastre et al. 1991; Moes et al. 2011). Since the apical bud is rich in F-actin, we asked whether it was also enriched in activated proteins implicated in signaling pathways. To address this issue, endothelial cells were analyzed at the early phases of spreading by staining with antibodies directed to proteins phosphorylated on tyrosine residues. Surprisingly, tyrosine-phosphorylated proteins were highly concentrated in the inner side of the apical bud, whose boundary was defined by Moesin labeling (Fig. 7a, b, not treated). To investigate the effects on tyrosine-phosphorylated proteins following loss of the actin network integrity, we treated endothelial cells with latrunculin B. Immunofluorescence analysis of cells fixed at various stages of early adhesion showed that the morphology of drug-treated adhering cells was severely affected and in these cells tyrosine-phosphorylated proteins were distributed throughout the cell without association with the aberrant Moesin-positive domains (Fig. 7a, b).

**Fig. 8:**
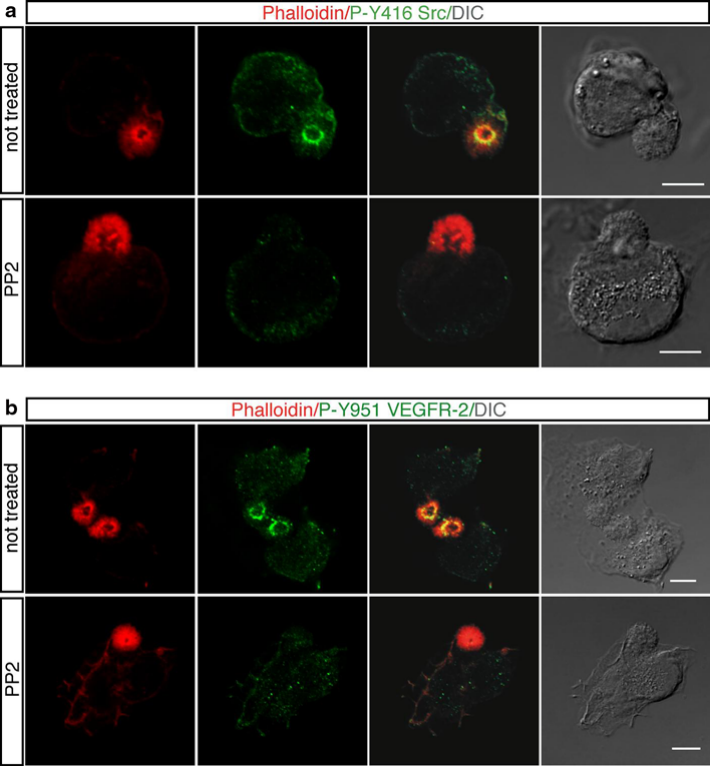
In adhering cells, activated Src and VEGFR-2 are localized in the core of apical actin domains. HUVEC were pretreated or not for 30 min with the Src inhibitor PP2 (10 μM), trypsinized, resuspended in complete medium, plated on vitronectin coat in the presence or not of the inhibitor, and fixed at different times after plating. **a** The early stages of cell spreading were analyzed by using phalloidin (*red*) and anti-phospho-Y416 Src antibodies (*green*). **b** Spreading cells were stained for F-actin (*red*) and phospho-Y951 VEGFR-2 (*green*). Treatment with PP2 affects phosphorylation of VEGFR-2 Y951 in the apical domain. DIC images of stained cells are shown. *Scale bars* represent 7 μm

Next, we investigated the activation of specific signaling molecules. Since activated Src is a tyrosine kinase required for rapid actin reorganization after early cell spreading (Partridge and Marcantonio 2006), we assessed the phosphorylation state of Src in adhering cells. As shown in Fig. 8a (not treated), phosphorylation on Src Y416, a protein modification that is closely correlated with kinase activity, was localized, in addition to discrete spots corresponding to peripheral adhesions, in the apical buds of spreading cells and clearly colocalized with F-actin in the core region as shown by the merged images. Then, we considered another signaling protein, the VEGF receptor-2 (VEGFR-2), which is implicated in all aspects of vascular-endothelial-cell biology (Ferrara et al. 2003; Olsson et al. 2006) and is positively regulated by plating endothelial cells onto vitronectin (Somanath et al. 2009). Interestingly, we observed that during the initial phases of cell spreading phosphorylation of the VEGFR-2 on Y951, a protein modification associated to actin reorganization (Matsumoto et al. 2005), displayed a pattern of distribution similar to active Src (Fig. 8b, not treated). Treatment with PP2, a selective Src inhibitor, affected phosphorylation of VEGFR-2 Y951 in the apical domain, suggesting that this specific receptor phosphorylation is Src-dependent (Fig. 8b, PP2). It is noteworthy that endothelial spreading cells treated with Src inhibitor displayed well-structured apical buds, indicating that the formation of the apical bud is independent of Src signaling (Fig. 8a, PP2). Since the VEGFR-2 can associate with VE-cadherin (Carmeliet et al. 1999; Lampugnani et al. 2006), we investigated the relocalization of VE-cadherin in VEGFR-2 silenced cells fixed at the early phases of spreading. Endothelial cells infected with lentiviruses expressing VEGFR-2 shRNA displayed well-structured and VE-cadherin-positive apical buds on their surface (Online Resource Fig. S5b, c), suggesting that the VEGFR-2 is not required for apical bud formation or for the relocalization of VE-cadherin to this cell structure. Thus, during cell attachment signaling molecules are activated in the core of apical buds and apical domain disruption leads to mislocalization of phosphorylated proteins. In addition, Src and the VEGFR-2 are associated to F-actin in the core of apical actin domains and VEGFR-2 activation depends on Src activity.

## Discussion

In this report, we describe the formation of a F-actin-rich domain on the apical membrane of endothelial cells, which is instrumental for two-dimensional polarization of spreading cells. We showed that rounded cells attaching to and spreading on the ECM modify their apical membrane forming a domain with a bud-like structure, which disappears when cells are fully flattened. Importantly, the actin-rich domain is formed on the membrane of postmitotic adhering cells and its formation does not depend on cell detachment treatment (enzymatic or biochemical), on the type of ECM substrate or on the presence of growth factors in the culture medium. Besides endothelial cells, different mammalian cell types form apical actin-rich domains during the initial phase of adhesion to the substrate. Cancer cells frequently lose apical–basal polarity, but in some cases cell polarization seems to play a role in metastatic spread during cancer invasion (Serrels et al. 2010). Accordingly, we observed that during spreading some types of cancer cells did not display apical actin domains, while others form apical domains variable in size and shape. Asymmetric distribution of actin in the apical cortex was described in melanoma and intestinal epithelial cells as a characteristic of polarizing cells (Baas et al. 2004; Estecha et al. 2009). Taken together with our findings, these data suggest that the presence of an actin-rich domain in adhering cells is a general phenomenon of polarizing cells.

Recent studies both in vitro and in vivo models have highlighted the key role of VE-cadherin and Moesin in symmetry breaking and polarity maintenance in endothelial cells (Strilic et al. 2009; Lampugnani et al. 2010; Wang et al. 2010). Here, we show polar distribution of VE-cadherin and Moesin in the apical bud during early endothelial cell adhesion, in agreement with a model positing that the role of these proteins is to promote formation of a polarized membrane surface. Both VE-cadherin and Moesin showed a similar pattern of colocalization with F-actin in the apical bud. This result is consistent with previous observations indicating that interaction of VE-cadherin with the actin cytoskeleton is relevant in the maintenance of cell polarity and that Moesin links specific membrane proteins to F-actin during cell-shape determination (Dejana et al. 2009; Fehon et al. 2010). However, to bind F-actin and membrane proteins, Moesin must be first activated by phosphorylation on a conserved threonine. Consistent with this requirement, we showed that Moesin colocalizing with F-actin in the apical bud was phosphorylated on T558. Furthermore, we found that Par3, a well-known polarity regulator involved in the establishment, maintenance, and transduction of polarity both in epithelial and endothelial cells (St Johnston and Ahringer 2010; Zovein et al. 2010), specifically localized to the core of the apical bud during the early phases of endothelial cell adhesion. Collectively, these results suggest that the apical actin-rich domain is a cell structure involved in the regulation of cell polarity during cell spreading.

Moesin has been implicated not only in cell-shape determination but also in upstream regulation of signal transduction (Strilic et al. 2009; Wang et al. 2010). Activated Moesin can potentially link specific membrane proteins to F-actin and bring receptors together with downstream components (Fehon et al. 2010). Moreover, the actin network has been demonstrated to be involved in signal transduction and in binding of receptors (Payrastre et al. 1991; Moes et al. 2011). Consistent with these observations, we found that following cell adhesion proteins phosphorylated on tyrosine were mainly localized in the apical bud and associated to F-actin, suggesting that the apical bud exhibits high levels of signaling transduction activity. We found that in the apical bud of adhering cells VEGFR-2 phosphorylation on Y951 was dependent on Src kinase activity. This result is consistent with our previous report demonstrating the adhesion-dependent phosphorylation of the VEGF receptor-3 by Src kinase (Galvagni et al. 2010). Src is involved in actin reorganization and specifically triggers apical plasma membrane remodeling in polarized epithelial cells (Mettlen et al. 2006). Thus, in its apical location, Src may modulate peripheral actin structures across cells to promote polarity. Indeed, phosphorylation of the VEGFR-2 on Y951 is a protein modification tightly associated to actin reorganization (Matsumoto et al. 2005). Collectively, these results suggest that in an attaching cell the cytoskeletal network in the apical domain might restrict membrane-protein diffusion throughout the cell and provide a simple mechanism for a local signaling centre to facilitate the formation of a polarized cell.

Crosstalk between the actin and microtubule cytoskeletons plays a crucial role during the initiation and maintenance of cell polarity. Actin appears to have a key role in the establishment of cell polarity enabling a rapid response to polarity cues, while microtubules stabilize the initial asymmetry created by actin-based forces by assisting the localization of key regulatory proteins to specific cortical sites (Li and Gundersen 2008). Microtubule features required to achieve this function are: orientation from the centrosome, post-translational modifications of tubulin, and protein transport (Li and Gundersen 2008; Quinones et al. 2011). Our study supports this concept of cytoskeletal regulation in the establishment and maintenance of cell polarity. By using inhibiting drugs and RNA interference technology we showed that while disruption of microtubules had no effects on apical domain structure, actin depolymerization and Moesin knockdown affected apical bud formation and the morphology of spreading cells, suggesting that Moesin and the actin network are responsible for the correct positioning of the apical bud in response to cell adhesion and strengthening the key role for Moesin in the regulation of structure and function of specific domains on the cell cortex (Fehon et al. 2010). Moreover, we identified an apical–basal axis of polarity, characterized by acetylated tubulin, from centrosome to apical bud that may serve as the tracks for the sorting of polarity proteins. We found that at the initiation of spreading apical polarity proteins, such as PODXL and caveolin-1, were localized along the apical–basal axis formed by acetylated tubulin, suggesting that microtubules are involved in the localization of key molecules to specific cortical sites. The presence of endosomal markers such as the transferrin receptor and the Rab5 and Rab11 GTPases along the acetylated tubulin tracks suggests that their redistribution may occur through polarized vesicular transport. The cellular function of PODXL and caveolin-1 during two-dimensional polarization remains unclear, but some mechanisms should be considered. Apical localization of PODXL may help cell to drive integrins to the basolateral surface of cells, essentially regulating expansion of the apical surface (Nielsen and McNagny 2008), whereas apical localization of caveolin-1, which interacts with proteins and cytoskeleton, may regulate plasma membrane organization and cell surface signaling (Parat et al. 2003; Lajoie et al. 2009).

Characteristics of polarized cells are: adhesion receptors that provide cues to orient cells by detecting the ECM, sorting of proteins to different plasma membrane domains, and signaling complexes differentially associated that define the biochemical features of resulting domains (Mellman and Nelson 2008). Although speculative, we propose a molecular mechanism of how endothelial cells attach and spread on ECM. When individual round cells adhere on the substrate, they derive spatial cues from integrin signaling, which induces a cortical symmetry-breaking event producing Moesin, VE-cadherin and Par3 polarity. Cells assemble both a basal domain and a Moesin-dependent apical domain in which different signaling proteins are clustered. The VEGF activation in the apical domain via Src kinase may induce the reorganization of the cytoskeleton. This polarization is further transmitted to the internal cell organization with the centrosome-apical bud axis oriented toward the apical surface for the sorting of polarity proteins, such as PODXL and caveolin-1. In conclusion, our results suggest that, without ruling it out as a possible site for growth factor internalization, the apical bud formed on the membrane of round cells during adhesion to the substrate represents a physical marker that, by local signaling pathways, orchestrates the cytoskeleton reorganization and establishes functionally distinct domains of the plasma membrane to develop an apical–basal axis of polarity.

## Electronic supplementary material

Below is the link to the electronic supplementary material.

Supplementary material 1 (MOV 1095 kb)

Supplementary material 2 (MOV 2141 kb)

Supplementary material 3 (MOV 2159 kb)

Supplementary material 4 (PDF 809 kb)

## Abbreviations

HUVEC: Human umbilical vein endothelial cells
PODXL: Podocalyxin
ECM: Extracellular matrix
VE-cadherin: Vascular endothelial cadherin
F-actin: Filamentous actin
VEGFR-2: Vascular endothelial growth factor receptor-2
Par3: Partitioning defective 3
