# Supplementary material for: An apical actin-rich domain drives the establishment of cell polarity during cell adhesion
Source: Histochem Cell Biol. 2012 May 29;138(3):419–33. doi: 10.1007/s00418-012-0965-9 (PMC3426669; doi:10.1007/s00418-012-0965-9)
Supplement: Supplementary file 4 — Supplementary material 4 (PDF 809 kb) [file 418_2012_965_MOESM4_ESM.pdf]

Supplementary material

An apical actin-rich domain drives the establishment of cell polarity during cell adhesion

Federico Galvagni, Cosima Tatiana Baldari, Salvatore Oliviero and Maurizio Orlandini

Corresponding author: Maurizio Orlandini, Dipartimento di Biotecnologie, Università degli Studi di Siena, Via Fiorentina 1 - 53100 Siena, Italy; E-mail: maurizio.orlandini@unisi.it.

Figure legends

Movie 1 Animation sequences of a *z*-series of transparent projections showing a primary endothelial cell adhering to vitronectin. Cells were fixed at 2 min after plating and stained with phalloidin. The movie shows a hemispheric cell with blebs at the periphery and a flat cell edge adhering to the substrate. A full-turn along the *x* axis is shown. The images in Fig. 1b (2 min) were derived from the same periodic *z*-series used for the animation of this movie

Movie 2 Animation sequences of a *z*-series of transparent projections showing a primary endothelial cell adhering to vitronectin. Cells were fixed at 25 min after plating and stained with phalloidin. The movie shows a spreading cell with extended lamellae, a flat cell body and the presence in its apical surface of an actin-rich domain. A full-turn along the *x* axis is shown

Movie 3 Animation sequences of a *z*-series of color-coded projections of a primary endothelial cell adhering to vitronectin. Cells were fixed at 25 min after plating and stained with phalloidin. The movie shows a spreading cell as in movie 2 with a violet basal cell surface, which adheres to the substratum, and a red apical surface, where an actin-rich domain is located. A full-turn along the *x* axis is shown. The images in Fig. 1b (25 min) were derived from the same periodic *z*-series used for the animation of this movie.

Fig. S1 Trypsin cell treatment, ECM proteins, and growth factor deprivation do not affect apical bud formation in spreading endothelial cells. (**a**) Growing HUVEC were washed with PBS, detached from the substratum using PBS containing 10 mM EDTA, washed, resuspended in complete medium and plated on vitronectin-coated coverslips. Scale bar represents 10 µm. (**b**) Cells were trypsinized, resuspended in complete medium and plated on glass coverslips coated with different ~~ECM~~ adhesive ~~proteins~~ molecules. Scale bars are 8 µm. (**c**) Growing cells were washed with PBS and maintained in starvation medium (M199 containing 0.1% foetal calf serum and 0.25% bovine serum albumin) for 5 h. Cells were then detached, resuspended in starvation medium and plated on vitronectin-coated coverslips. Scale bar is 6 µm. Spreading cells were stained with phalloidin (green). Arrowheads indicate the actin-rich domain. DIC images of the same cells are shown.

Fig. S2 Moesin accumulates in the actin-rich domains of postmitotic spreading endothelial cells. HUVEC were plated on coverslips coated with vitronectin. 2 h after plating, culture medium was removed, cells washed with PBS and fresh medium added. 20 h after medium removal, cells were stained for F-actin (green), Moesin (red) and DNA (blue). Scale bars are 8 µm. The upper panels show the same cells of Fig. 1D

Fig. S3 During cell spreading PODXL and caveolin-1 localize to the internal side of the apical bud. (**a**) Details of a spreading endothelial cell. Cells were seeded on vitronectin-coated coverslips and fixed at different times.

Cells were stained using anti-Moesin (red) and anti-PODXL (green) antibodies. The actin bud is marked by Moesin staining. Scale bar is 3 μm. (**b**) Endothelial cells were treated or not for 30 min with latrunculin B. Then they were trypsinized, resuspended in complete medium and plated on vitronectin in the presence or not of latrunculin B. Cells were imaged as in **a**. Periodic *z*-series of spreading cells were acquired at 20 min and the corresponding lateral view (top panel) and a white dotted line indicating cell boundary (bottom panel) are shown. Scale bars represent 6 μm. (**c**) Details of a spreading endothelial cell. Cells treated as in **a** were stained for F-actin (green) and caveolin-1 (red). Phalloidin staining marks the apical bud. Scale bar is 3 μm

Fig. S4 A protein trafficking route is located along the centrosome-apical domain axis during cell spreading. Endothelial cells were seeded on vitronectin-coated coverslips and fixed at different spreading times. (**a**) Cells were stained for Moesin (red) and Centrin 1 (green), a protein involved in the determination of centrosome position and segregation. An arrowhead indicates the centrosome localized above the nucleus. A white dotted line indicates nucleus location. Scale bar is 6 μm. (**b**) Cells were stained for Moesin (red) and Rab5 or Rab11 (green). Scale bar represent 6 μm. The figures show cells fixed after 5 min from plating. DIC image and merge are shown

Fig. S5 VE-cadherin localization in latrunculin B-treated and VEGFR-2-silenced spreading endothelial cells. (**a**) ~~Endothelial~~ Cells were pretreated for 30 min with latrunculin B, then were trypsinized, resuspended in complete medium and plated on vitronectin in the presence of latrunculin B. Adhering cells were fixed and stained for F-actin (green) and VE-cadherin (red). VE-cadherin mislocalizes to several cytosolic regions. Scale bar is 13 μm. (**b**) Cells were infected with a lentiviral vector expressing unrelated (unr) or VEGFR-2 (clone 85 and clone 86) shRNA. Cell extracts from HUVEC expressing shRNA were analyzed by Western blotting using anti-VEGFR-2 and anti-β-actin antibodies. Cells infected with lentiviruses expressing either VEGFR-2 shRNA show a reduction in VEGFR-2 protein expression with respect to cells infected with a lentivirus expressing an unrelated shRNA. (**c**) Endothelial cells infected as in **b** were detached from the plate, seeded on vitronectin-coated coverslips and fixed at different times after plating. Cells were stained for F-actin (green) and VE-cadherin (red). Every VEGFR-2-silenced cell forms the apical bud on its surface during spreading. A representative adhering cell is shown. Scale bar is 7 μm. DIC images of stained cells are shown

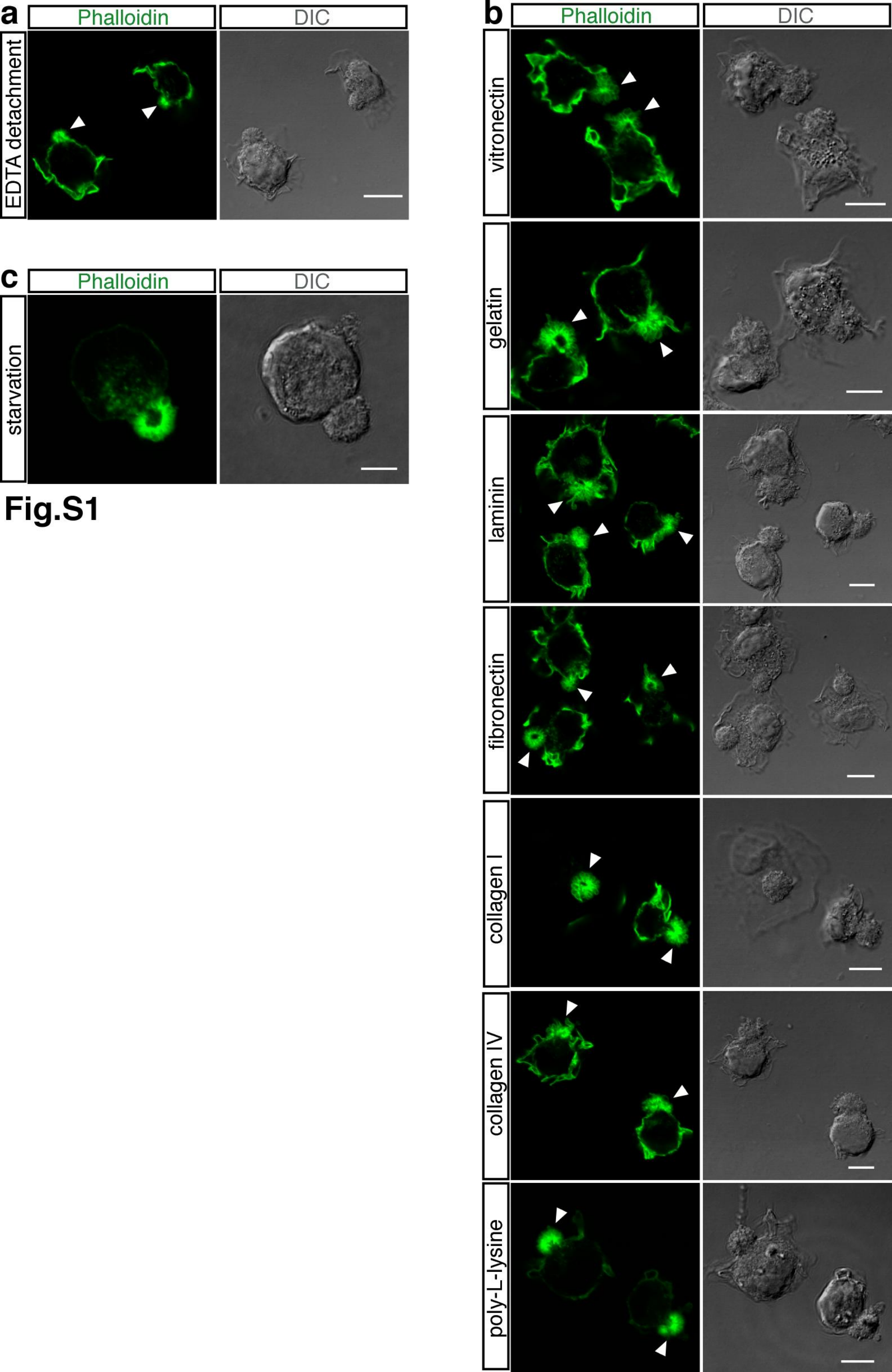
a
Phalloidin
DIC
EDTA detachment
b
Phalloidin
DIC
vitronectin
gelatin
laminin
fibronectin
collagen I
collagen IV
poly-L-lysine
c
Phalloidin
DIC
starvation

Fig.S1

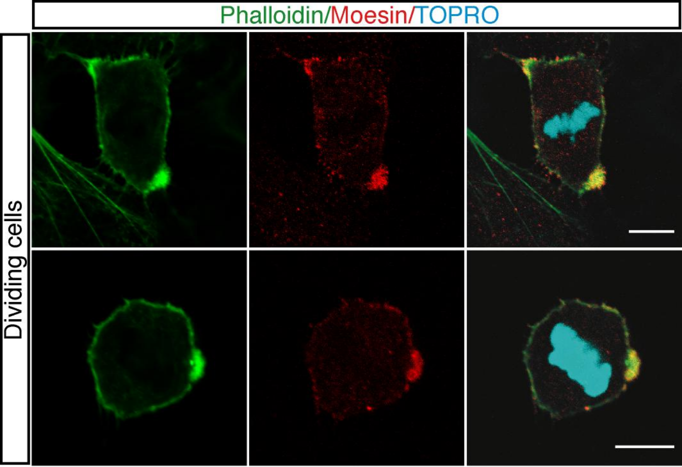

Fig. S2

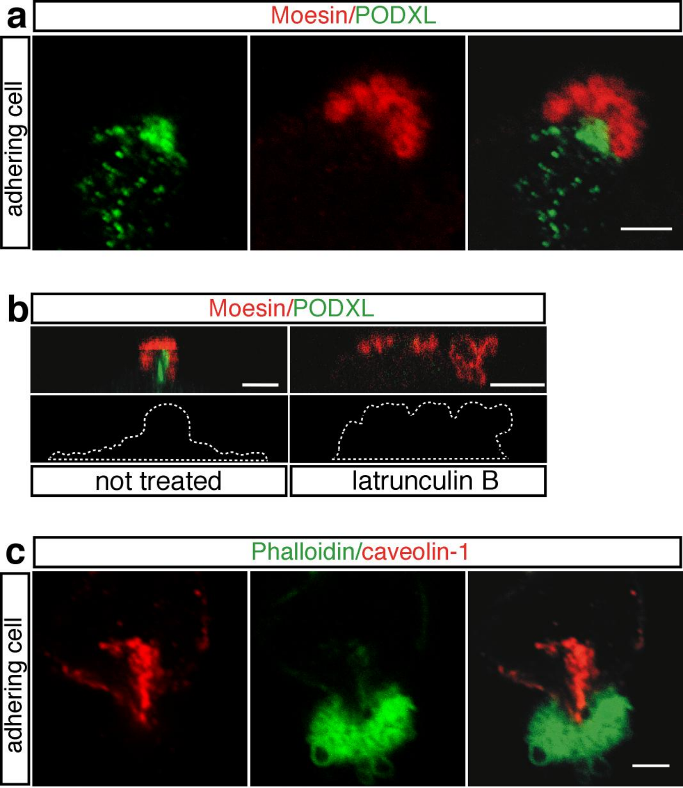
a
Moesin/PODXL
adhering cell
b
Moesin/PODXL
not treated
latrunculin B
c
Phalloidin/caveolin-1
adhering cell

Fig. S3

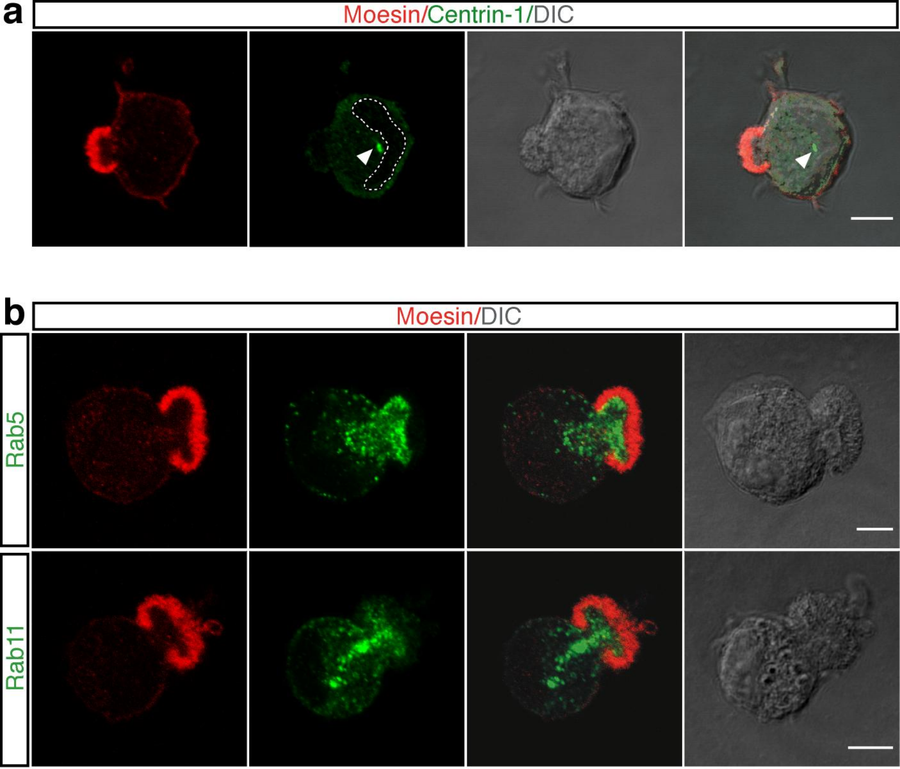

Fig. S4

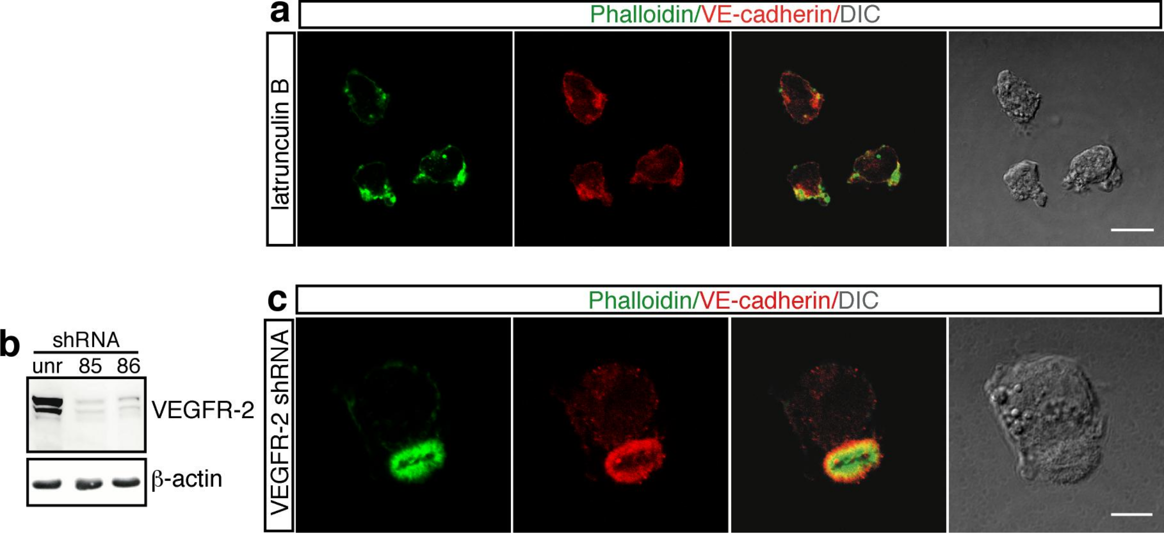
a
Phalloidin/VE-cadherin/DIC
latrunculin B
b
shRNA
unr
85
86
VEGFR-2
β-actin
c
Phalloidin/VE-cadherin/DIC
VEGFR-2 shRNA

Fig. S5
